# Effects of Cardiotoxins from *Naja oxiana* Cobra Venom on Rat Heart Muscle and Aorta: A Comparative Study of Toxin-Induced Contraction Mechanisms

**DOI:** 10.3390/toxins14020088

**Published:** 2022-01-24

**Authors:** Alexey S. Averin, Miroslav N. Nenov, Vladislav G. Starkov, Victor I. Tsetlin, Yuri N. Utkin

**Affiliations:** 1 Institute of Cell Biophysics of the Russian Academy of Sciences, Federal Research Center “Pushchino Scientific Center of Biological Research of the Russian Academy of Sciences”, 142290 Pushchino, Russia; averinas82@gmail.com; 2 Institute of Theoretical and Experimental Biophysics, Russian Academy of Sciences, 142290 Pushchino, Russia; nenovmir@gmail.com; 3 Shemyakin-Ovchinnikov Institute of Bioorganic Chemistry, Russian Academy of Sciences, 117997 Moscow, Russia; vladislavstarkov@mail.ru (V.G.S.); victortsetlin3f@gmail.com (V.I.T.)

**Keywords:** cardiotoxin, heart, papillary muscle, aorta, contraction, contracture

## Abstract

Cardiotoxins (CaTxs) are a group of snake toxins that affect the cardiovascular system (CVS). Two types (S and P) of CaTxs are known, but the exact differences in the effects of these types on CVS have not been thoroughly studied. We investigated cellular mechanisms of action on CVS for *Naja oxiana* cobra CaTxs CTX-1 (S-type) and CTX-2 (P-type) focusing on the papillary muscle (PM) contractility and contraction of aortic rings (AR) supplemented by pharmacological analysis. It was found that CTX-1 and CTX-2 exerted dose-dependent effects manifested in PM contracture and AR contraction. CTX-2 impaired functions of PM and AR more strongly than CTX-1. Effects of CaTxs on PM were significantly reduced by nifedipine, an L-type Ca^2+^ channel blocker, and by KB-R7943, an inhibitor of reverse-mode Na^+^/Ca^2+^ exchange. Furthermore, 2-aminoethoxydiphenyl borate, an inhibitor of store-operated calcium entry, partially restored PM contractility damaged by CaTxs. The CaTx influence on AR contracture was significantly reduced by nifedipine and KB-R7943. The involvement of reverse-mode Na^+^/Ca^2+^ exchange in the effect of CaTxs on the rat aorta was shown for the first time. The results obtained indicate that CaTx effects on CVS are mainly associated with disturbance of transporting systems responsible for the Ca^2+^ influx.

## 1. Introduction

The cardiovascular system (CVS) is one of the main targets of snake venom toxins. Although a significant number of venoms affect the activity of the CVS, not all of them act directly on the heart. For example, the venom of the Papuan taipan (*Oxyuranus scutellatus*), which causes cardiovascular collapse in anaesthetized rats, does not affect the function of an isolated heart [1]. In general, the number of snake toxins that have a direct effect on the heart and blood vessels is relatively small. Thus, recently, it was shown that phospholipases A_2_ from venoms of *Pseudonaja* genus snakes are involved in vasorelaxation [2]. Further, cobra venom cytotoxins, also called cardiotoxins [3,4] are well known for their direct cardiovascular effects.

Cardiotoxins (CaTx) from cobra venoms are β-structured proteins consisting of 59–61 amino acid residues with four disulfide bonds [5]. Upon entering the organism, CaTxs primarily have a damaging effect on the CVS, causing systolic cardiac arrest [6]. On preparation of the heart muscle, it was shown that CaTxs produced a decrease in contractility, depolarization, and contracture [7]. Most CaTxs act directly on cardiac tissue in both the ventricles and the atria without showing any selectivity. However, there are tissue-specific features in the development of effects—some CaTxs cause more pronounced action on the ventricular tissue than others [7]. Previous studies on the mechanism of CaTx action showed that, in cardiomyocytes, these toxins induce an increase in the concentration of calcium ions inside the cell, a change in the cell shape, hypercontracture, and death [8]. Recently, it was shown that CaTxs are able to penetrate not only through the cell plasma membrane, but also through the outer mitochondrial membrane of the cell [9]. They then target anionic cardiolipin and disrupt the structure of the inner mitochondrial membrane and bioenergetics, which can lead to the death of cardiomyocytes [9,10]. This may represent the complementary pathway for CaTx toxicity.

CaTxs are classified into S- and P-types. The S-type includes toxins with a serine residue at position 28 of the amino acid sequence, while the P-type comprises those with a proline residue at position 30 [5]. The mechanism of interaction of CaTxs with lipid membranes depends on the type of toxin [11]. The data available to date indicate that toxins of both types destabilize the lipid bilayer of anionic phospholipid-containing membranes, but with different efficacy [11]. However, we were unable to find data on the effect of different types of toxins on the heart muscle and blood vessels. It should be noted that researchers did not provide exact data on the amino acid sequence of the CaTx used in all cases. However, in those studies that provided precise indications of the structure of the CaTx, the toxin CTX A3 of the P-type from the venom of the cobra *Naja atra* was typically used (e.g., [12]).

Thus, a comparative analysis of the activity of CaTxs and the mechanisms of the development of their effects in time remains an unsolved problem. To make such a comparison, in this work we study the effects of cytotoxins I and II (CTX-1 and CTX-2), belonging to the S- and P-types, respectively, from the venom of the Central Asian cobra *Naja oxiana* [13] on the contractility of rat myocardial and thoracic aorta preparations, the latter preparation being used as a sample of smooth muscle tissue. In addition, the question of the effects of sub-threshold, non-contracture CaTx concentrations over several hours of application remains unclear. On the one hand, significant changes in the level of expression of individual genes can already occur over such a time interval [14]; on the other hand, specific interactions with any of the components of signaling systems may occur [15,16,17]. Thus, our goal was to carry out a comparative study of two CaTxs of different types on two different types of muscle tissue to compare their activity on different types of tissue present in the CVS. We previously investigated the effect of CaTxs on the rat papillary muscles (PMs) and found that two cobra *Naja oxiana* CaTxs changed the force of contraction and the character of rhythmoinotropic phenomena in the rat myocardium [18]. Here, we carried out a more detailed study by conducting a comparative analysis of the effects of CaTxs on both the rat myocardium PM and on the descending segment of the rat aorta. We also carried out an extensive pharmacological analysis in order to shed light on the possible role of various Ca^2+^ transport systems in the effects of CaTxs on the contractile activity of the PM and aorta ring (AR).

## 2. Results and Discussion

**Figure 1:**
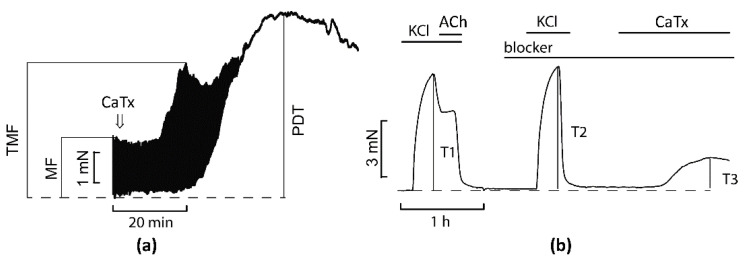
Schematic illustration of the experiments on contractility of papillary muscles (PMs) (**a**) and contraction of aorta rings (ARs) (**b**). MF, maximal force before application of CaTx; TMF, transient maximal force after CaTx application; PDT, peak diastolic tension (contracture) developed in response to CaTx application; KCl, isotonic solution with 80 mM KCl; ACh, 10 µM acetylcholine; CaTx, cardiotoxin; blocker, a pharmacological inhibitor of particular Ca^2+^ transporting system. Arrow on the panel (**a**) indicates CaTx application. T1, tension at 80 mM KCl; T2, tension at 80 mM KCl in the presence of blocker; T3, tension in the presence of both blocker and CaTx.

A schematic illustration explaining the terms used below and some experimental details is shown in Figure 1 and Figure S1.

### 2.1. Comparison of Cardiotoxins Effects on the Rat Papillary Muscle and Aorta

It was previously shown that CaTxs act on cardiac tissue in two ways, causing both a decrease in contractility and a development of contracture [19,20]. In blood vessels, CaTxs induce a slowly developing tonic contraction, while in the ARs precontracted by phenylephrine, a transient relaxation effect caused by the activation of endothelial cells was observed in the presence of CaTxs [21]. 

**Figure 2:**
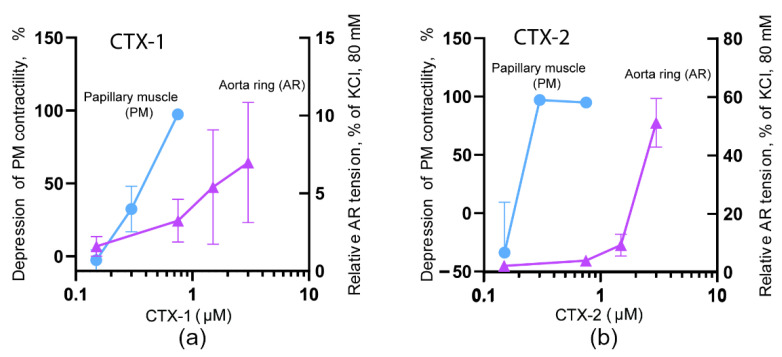
Dependence of papillary muscle (PM) and aorta ring (AR) contractions on concentration of CTX-1 (**a**) and CTX-2 (**b**). The inhibition of PM contraction by CaTxs as compared to the control level is shown on the left ordinate (blue circles). The contraction of AR induced by CaTxs compared to 80 mM KCl is shown on the right ordinate (pink triangles). The effects were registered 1 h after toxin application.

In our experiments, CTX-1 at a concentration of 0.75 µM produced a complete suppression of PM contraction (by 97 ± 2%, Figure 2a), while in the ARs a concentration of 3 µM induced a fairly small contractile response (of 7 ± 4%, Figure 2a). At the same time, CTX-2 at a concentration of 0.3 µM completely suppressed PM contractility (by 96 ± 5%), while a concentration of 3 µM induced a contraction of AR equal to 51 ± 8% (Figure 2b). These experiments showed that in order to achieve the maximal effect on AR, both CTX-1 of S-type and CTX-2 of P-type need an order of magnitude higher concentration than that required for maximal effect on PM. In general, this is in good agreement with the available data; for example, in studies on smooth muscle preparations including ARs [21,22] and intestinal strips [23], CaTx concentrations of about 10 µM were used. Further, in studies on myocardial preparations, CaTx concentrations of about 1 µM or less were usually employed [8,24]. However, there are some differences in the description of the CaTx effects on various types of tissue. There is evidence that on some smooth muscle preparations, a full contractile response develops at a concentration of slightly more than 1 µM [25], which is an order of magnitude less than the concentration described in most existing works. CaTxs can also differ significantly in their effect on the myocardium: on the one hand, there are CaTxs that can induce contracture at concentrations of less than 1 μM, while others, even at a concentration of more than 10 μM, do not lead to the development of contracture [24]. Prior to our present work, there were no studies comparing the effects of the two types of CaTxs on myocardial and smooth muscle tissues. 

It should be noted that the differences in the effects of CaTxs are most pronounced at sufficiently low concentrations [18,24], while at high toxin concentrations of about 10 μg/mL, the difference may not be observed [26]. Our results, obtained at low concentrations of toxins, concluded that CTX-2 possesses higher activity than CTX-1 both on PM and AR preparations, which significantly supplements the previously obtained data on a more pronounced effect of CTX-2 on heart preparations [13]. Interestingly, recently the difference in activity between CaTxs of P- and S-types was observed on cancer cell lines [27]. It was shown that on human lung (A549), prostate (PC-3), and breast (MCF-7) P-type CaTx NS-CTX was significantly more potent in inhibiting the growth of the cancer cells than S-type CaTx NK-CTX.

### 2.2. Comparison of Cardiotoxins Time-Dependent Effects on Contractility and Diastolic Tension in Rat Papillary Muscle

**Figure 3:**
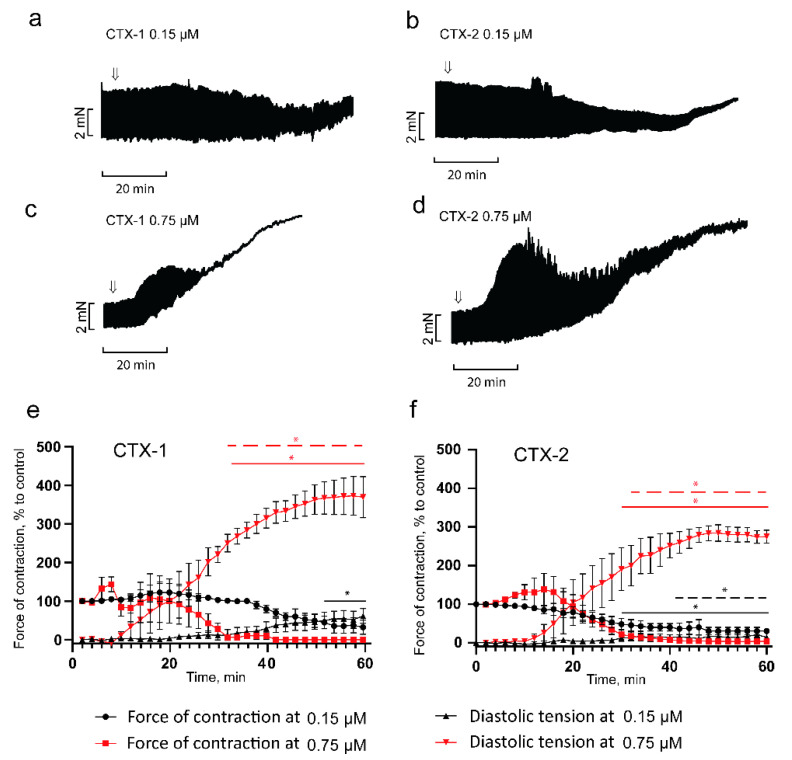
Influence of CTX-1 (S-type) and CTX-2 (P-type) on isometric contraction force and diastolic tension of the right ventricle papillary muscle. (**a**,**c**): representative traces showing the effects of CTX-1 at concentrations of 0.15 (*n* = 4) and 0.75 (*n* = 3) μM. (**b**,**d**): representative traces showing the effects of CTX-2 at concentrations of 0.15 and 0.75 μM (*n* = 4 for both). Arrows indicate an application of toxins. The stimulation frequency is 0.3 Hz. (**e**,**f**): typical examples for the development of the effect of CTX-1 and CTX-2 over time. The ordinate shows the force of contraction (rings and squares) or diastolic tension (triangles) normalized to the force of contraction obtained at 0.3 Hz in the control. Data are presented as mean ± SEM (* *p* < 0.05). Horizontal solid lines represent significant difference in papillary muscle contraction force, and dashed lines represent significant difference in papillary muscle diastolic tension. Black and red lines indicate toxin concentration of 0.15 and 0.75 µM, respectively.

Using relatively low concentrations of CaTxs, we investigated the development of their effects over time (Figure 3). It was found that CTX-1 at a concentration of 0.15 μM produced a progressive negative inotropic effect, which after 52 min of exposure became significant, reducing contractility to 45 ± 16% of the control values (Figure 3a), and by 60 min the force of contraction decreased to 32 ± 19% of control. A similar effect was manifested by CTX-2 at the same concentration; however, a significant decrease in contractility occurred as early as by 28 min (53 ± 15% of control), which is almost twice as fast as the observed decrease of CTX-1. By 60 min, contractility in the presence of CTX-2 decreased to 21 ± 7% of control (Figure 3b). It should be noted that in some experiments, both toxins produced a transient increase in contractility, which was then followed by suppression.

When studying the effect of CaTXs on diastolic tension, we found that at 0.15 μM, CTX-1 produced a gradually increasing diastolic tension, reaching 62 ± 19% by 60 min after toxin application (Figure 3a,e). An increase in diastolic tension in the presence of 0.15 μM CTX-2 became significant at 42 min (26 ± 7%), reaching 40 ± 17% by 60 min (Figure 3b,f). In the presence of higher concentrations of toxins (0.75 μM), the effects developed much faster and were more pronounced. At a CTX-1 concentration of 0.75 μM, a transient increase in the force of contractions up to 132 ± 21% of the control was observed after 8 min, then the contractility decreased, and after 32 min it reached 8 ± 5% of the control; it took more than 50 min to achieve the maximal contracture, which was equal to 369 ± 53% of control (Figure 3e). Under the action of CTX-2, a transient increase in the force of contractions reaching 138 ± 41% of the control developed within 20 min, then the contractility decreased, and after 38 min it was equal to 9 ± 7% of the control; more than 50 min were required to reach the maximum contracture level of 274 ± 16% (Figure 3f). After the development of contracture induced by either toxin, the effect could not be reversed by washing off the toxin (data not shown). It should be noted that the more rapid suppression of contractions by the CTX-2 at 0.15 μM confirmed the previously obtained data on the higher activity of this toxin as compared to CTX-1 [18]. At the same time, at a concentration of 0.75 μM, the difference between toxins was minimal, as CTX-2 induces a slightly more pronounced transient increase in the contraction force at the onset of the action, which may indicate that concentration of 0.75 μM was already close to causing saturation. These data suggest that CTX-2, in comparison to CTX-1, has a more pronounced effect on the contractility and resting tension of myocardial tissue. Both toxins at concentration of 0.75 µM exert similar time-dependent effect on the development of PM contracture and show the similar activity level (Figure 3e,f).

It should be noted that some studies also report a positive inotropic response to the application of CaTxs [28]. As a rule, the effect is recorded in a fairly narrow concentration range, is transient, and is followed by the development of contracture with suppression of contractile activity. While for most CaTxs the contracture-inducing concentration ranges from 1 to 10 μg/mL [20,24], there are toxins that produce a positive inotropic response and do not induce contracture at doses up to 100 μg/mL [24]. The nature of this effect is not yet completely clear, although it has been suggested that the rate of release of calcium ions from the sarcoplasmic reticulum may increase [29]. 

### 2.3. Effects of Cardiotoxins on Force-Frequency Relationship in Papillary Muscle

Previously, it was shown that cobra venom produces the most pronounced changes in the expression level of gene encoding signals and ion transporting proteins in the heart [14]. However, there is evidence that long-term exposure to individual components of snake venom can change the expression level of key proteins essential for electromechanical coupling, significantly improving myocardial contractile function [30]. Until now it was not known what changes in the physiological response might result from prolonged exposure to low concentrations of CaTxs (not causing contracture), the key proteins of the cobra venom. 

**Figure 4:**
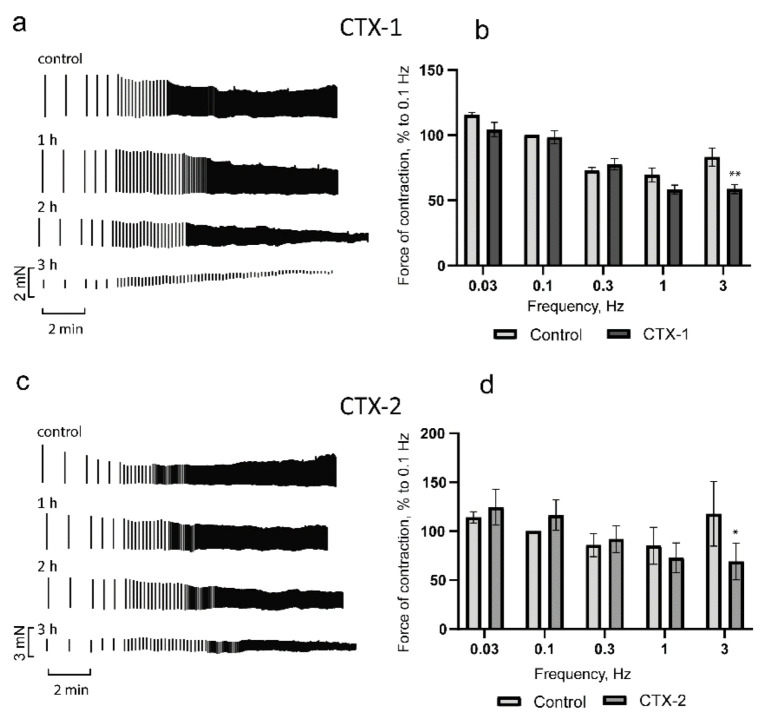
Influence of CTX-1 (0.15 µM) and CTX-2 (0.075 µM) on rat papillary muscle contractility. (**a**,**c**) are typical examples of the development of the effect over time for CTX-1 and CTX-2, respectively. The force–frequency relationship (FFR) data for (**b**) CTX-1 (*n* = 4) and (**d**) CTX-2 (*n* = 5). The ordinate shows the force of contraction, normalized to that obtained at a frequency of 0.1 Hz in the control. The abscissa shows the stimulation frequency in Hz. The data are shown for one hour of incubation with CaTxs and presented as means ± standard error of the mean (* *p* < 0.05; ** *p* < 0.01 compared to control).

Since in the study of the concentration dependence it was found that the minimum concentration of CTX-1 required to cause contracture within 30–60 min was 0.3 µM for CTX-1 and 0.15 µM for CTX-2. In further studies of the dependence of the PM contraction force on stimulation frequency, a two-fold lower concentration of 0.15 µM for CTX-1 and 0.075 µM for CTX-2 was used. At concentrations that did not induce contracture, the action profiles of the cardiotoxins were similar. In the frequency range of up to 1 Hz, a mixed picture was observed; the force of the contractions in comparison with the control decreased at some frequencies and increased at others, but the differences were not significant (Figure 4a,c). At a stimulation frequency of 3 Hz, both toxins produced significant reductions in contractile force of 30 ± 4% and 42 ± 5% for CTX-1 and CTX-2, respectively (Figure 4a,c). It should be noted that during the transition from 1 to 3 Hz, the contraction force decreased and the positive segment of the force–frequency relationship (FFR) was blocked. 

In the range of physiological frequencies, the myocardium of rats and mice is characterized by a positive FFR [31], the formation of which is led by the frequency-dependent activation of L-type Ca^2+^ current [32]. This regulation of current is one of the most sensitive molecular mechanisms of contractility regulation. With prolonged exposure to cardiotoxins, the FFR remained negative at all studied frequencies and the contraction strength progressively decreased (Figure 4). This may suggest the impairment of frequency-dependent activation of L-type Ca^2+^ current by CaTxs.

### 2.4. Molecular Mechanisms of the Papillary Muscle Contractile Response Induced by Cardiotoxins

**Figure 5:**
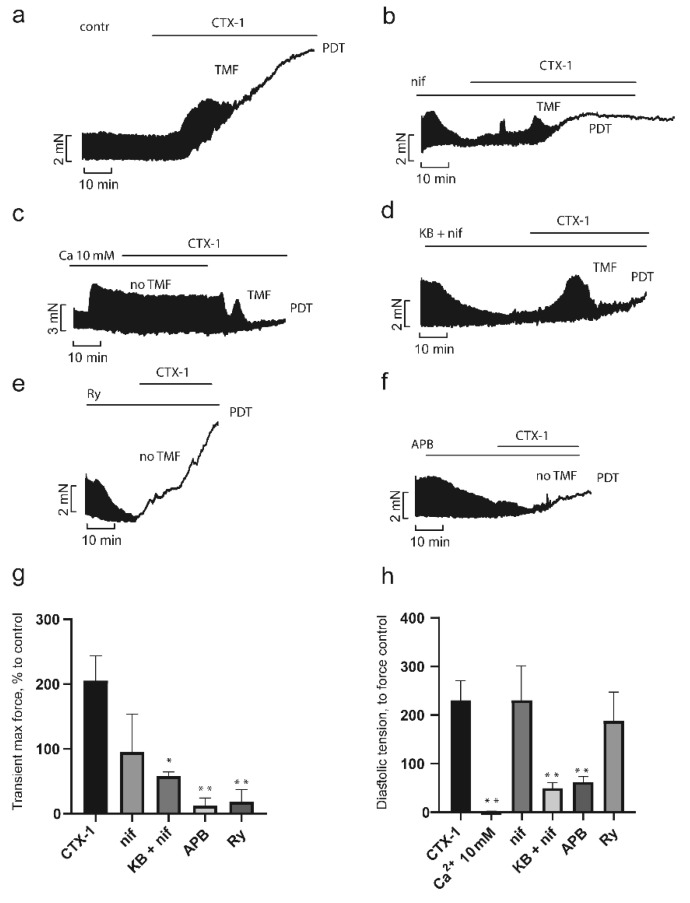
Influence of inhibitors of Ca^2+^ transport mechanisms on the CTX-1 effects in papillary muscle. Typical recordings of the development of the CTX-1 effect over time in control (**a**) (*n* = 5) and in the presence of inhibitors (**b**–**f**). CTX-1 effects on transient maximal force (TMF) (**g**) and diastolic tension (**h**) in the absence and in the presence of inhibitors. Data are presented as means ± standard error of the mean (* *p* < 0.05; ** *p* < 0.01 compared to the CTX-1 level). PDT, peak diastolic tension (contracture); Ca, 10 mM Ca^2+^ (*n* = 3); Ry, ryanodine (1 µM, *n* = 4); nif, nifedipine (2 µM, *n* = 4); KB + nif, KB-R7943 (10 µM) + nifedipine (2 µM) (*n* = 3); APB, 2-aminoethoxydiphenyl borate (10 µM, *n* = 4). The contraction force registered before application of inhibitor and CaTx was taken as a control.

**Figure 6:**
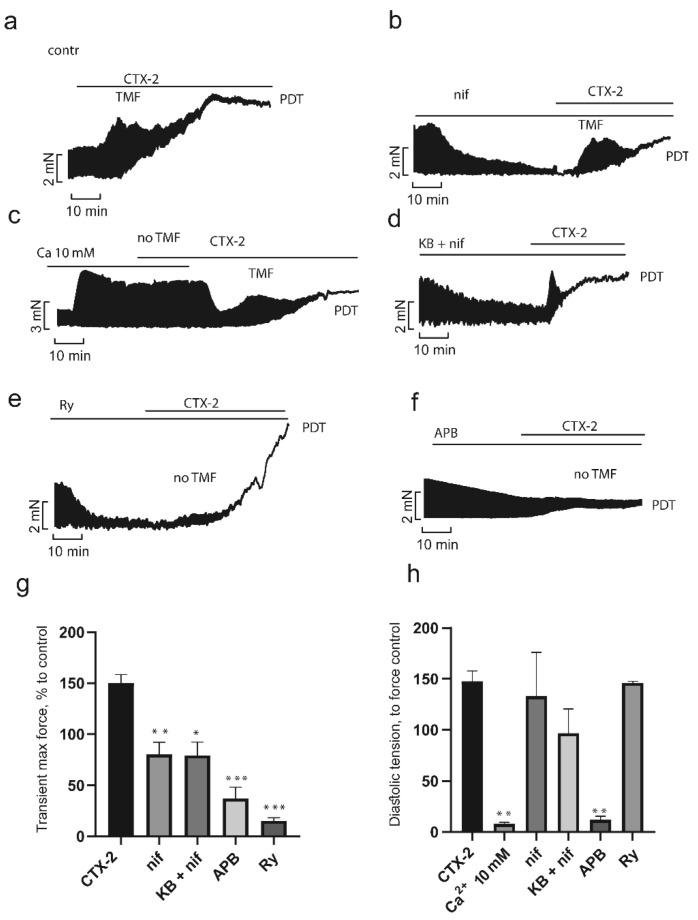
Influence of inhibitors of Ca^2+^ transport mechanisms on the CTX-2 effects in papillary muscle. Typical recordings of the development of the CTX-2 effect over time in control (**a**) (*n* = 5) and in the presence of inhibitors (**b**–**f**). CTX-2 effects on transient maximal force (TMF) (**g**) and diastolic tension (**h**) in the absence and in the presence of inhibitors. Data are presented as means ± standard error of the mean (* *p* < 0.05; ** *p* < 0.01; *** *p* < 0.005 compared to the CTX-2 level). PDT, peak diastolic tension (contracture); Ca, 10 mM Ca^2+^ (*n* = 3); Ry, ryanodine (1 µM, *n* = 4); nif, nifedipine (2 µM, *n* = 6); KB + nif, KB-R7943 (10 µM) + nifedipine (2 µM) (*n* = 4); APB, 2-aminoethoxydiphenyl borate (10 µM, *n* = 5). The contraction force registered before application of inhibitor and CaTx was taken as a control.

To study the role of various Ca^2+^ transport mechanisms in the development of the CaTx effects, we used concentrations of toxins, which within 30 min produced pronounced characteristic effects. Since the activity of each CaTx differs significantly, we employed concentration of 0.75 µM for CTX-1 and 0.3 µM for CTX-2. As seen in Figure 5a and Figure 6a, at the chosen concentrations CaTxs acted in a characteristic manner; a transient increase in contractility (TMF) 200 ± 16% and 150 ± 9% for CTX-1 and CTX-2, respectively, was followed by its inhibition, with a simultaneous increase in the maximum diastolic tension followed by the development of peak diastolic tension (PDT) of 225 ± 36% for CTX-1 and 148 ± 12% for CTX-2.

One of the key factors in the pathological effects of CaTxs is the overload of cells with Ca^2+^ ions. However, there is some controversy about the source of the increased intracellular concentration of Ca^2+^ ions. To clarify the source, we used different blockers of calcium channels and transporters. The release of Ca^2+^ ions from the sarcoplasmic reticulum (SR) is known to play a key role in the development of contractions [33]. To assess the role of the release of Ca^2+^ ions from the SR in the development of CaTx effects, we used ryanodine, a blocker of ryanodine-sensitive calcium channels. As seen in Figure 5e and Figure 6e, at the suppression of Ca^2+^ release from SR with ryanodine (1 µM), the rapid growth phase of contractility (TMF) in response to the administration of both CaTxs was almost completely blocked, being only 18 ± 9% and 15 ± 3% for CTX-1 and CTX-2, respectively (Figure 5g and Figure 6g). At the same time, the magnitude of contracture (increase in diastolic tension, PDT) did not differ from that observed in the absence of ryanodine and was equal to 188 ± 60% and 146 ± 2% for CTX-1 and CTX-2, respectively (Figure 5h and Figure 6h). The relationship between the transient increase in contractility in response to CaTxs, which we observed, can be a consequence of both an increase in Ca^2+^ release from the SR due to the accumulation of Ca^2+^ in the cytosol and an increase in the sensitivity of the ryanodine receptor (RyR) to Ca^2+^ under the influence of CaTx [34]. 

In our experiments, similarly to the literature data [8,24,26], a high calcium concentration (10 mM) prevented the development of contracture caused by CaTxs in PM preparations. As seen in Figure 5c,h as well as Figure 6c,h the contraction force and diastolic tension in the presence of 10 mM Ca^2+^ remained unchanged after CaTx application. 

One of the main paths of extracellular Ca^2+^ entry into the cell are L-type Ca^2+^ channels. In our experiments, PM pretreatment with L-type Ca^2+^ channel blocker nifedipine (2 μM) did not prevent the development of the effects of CTX-1 and CTX-2 (Figure 5b and Figure 6b). The transient growth, while remaining quite pronounced, decreased to 95 ± 29% and 80 ± 12% for CTX-1 and CTX-2, respectively (Figure 5g and Figure 6g). The magnitude of contracture did not change and was equal to 218 ± 74% and 133 ± 43% (Figure 5h and Figure 6h). Our data are consistent with most of the studies performed on myocardial preparations from various animal species, including the atria of the guinea pig [24] and the cardiomyocytes of adult rats [8]. However, it was also shown that the L-type Ca^2+^ channel blockers can prevent the development of contracture on neonatal rat cardiomyocytes [35] or reduce the magnitude of contracture on guinea pig PM [26]. In our experiments, both cardiotoxins retained the ability to induce contracture of rat PM preparations after pretreatment with nifedipine (2 μM). These differences can be explained by the fact that in the rat myocardium, the reverse-mode of Na^+^/Ca^2+^ exchange (NCX), as well as other types of channels, e.g., transient receptor potential channels, can provide an influx of Ca^2+^ ions sufficient to initiate the release of Ca^2+^ from the SR and develop contracture. To test this hypothesis, we studied the influence of simultaneous pretreatment of PM with nifedipine and a reverse-mode NCX blocker KB-R7943 (10 µM) on the CaTx effects. As seen in Figure 5d and Figure 6d, the TMF decreased slightly to 95 ± 29% for CTX-1 and 79 ± 13% for CTX-2 (Figure 5g and Figure 6g), while the magnitude of contracture decreased significantly to 50 ± 11% and 70 ± 3%, respectively (Figure 5h and Figure 6h). Thus, it can be concluded that both the L-type Ca^2+^ current and the reverse-mode NCX are involved in the development of CaTx-induced Ca^2+^ overload in the rat PM.

Store-operated calcium entry (SOCE) is another possible mechanism of entry for extracellular Ca^2+^ ions. To determine the role of this mechanism, we used 2-aminoethoxydiphenyl borate (2-APB, 10 µM), a non-selective SOCE blocker. As seen in Figure 5f,g as well as Figure 6f,g, after 2-APB pretreatment, the TMF significantly decreased to 12 ± 7% for CTX-1 and 37 ± 11% for CTX-2. Moreover, in a significant number of experiments, TMF did not occur at all. However, the fairly high level of TMF seen in Figure 5g and Figure 6g, especially for CTX-2, indicates incomplete inhibition of contraction in the presence of 2-APB. The magnitude of contracture was also reduced significantly to 48 ± 17% for CTX-1 and 10 ± 4% for CTX-2 (Figure 5h and Figure 6h). 

**Figure 7:**
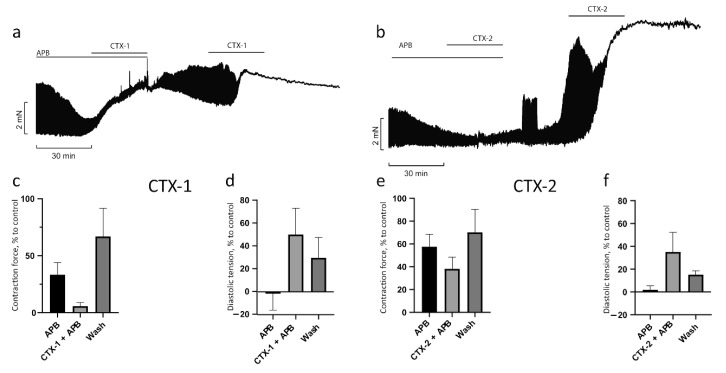
Preliminary blockade of SOCE with 2-APB allows the restoration of the contractile activity of papillary muscle after exposure to cardiotoxins. Typical recordings of the effect development over time after preincubation with 2-APB, washing out, and another application of CTX-1 (**a**) and CTX-2 (**b**). The effects on contraction force (**c**,**e**) for CTX-1 and CTX-2, respectively, as well as on diastolic tension (**d**,**f**) for CTX-1 and CTX-2, respectively (*n* = 3 for both toxins). Data are presented as means ± standard error of the mean.

Previously, in our study and the studies of others, it has been shown that the contracture produced by CaTxs was not reversed after washing out the toxin [18,24,36]. To determine whether 2-APB can reverse the effect of CaTxs, we carried out experiments that involved washing out the CaTx after exposure of the PM to 2-APB. As seen in Figure 7, in the presence of 2-APB, under the influence of CTX-1 (0.3 µM) and CTX-2 (0.15 µM), the contraction force decreased to 6 ± 3% and 36 ± 12%, respectively, of the control level (Figure 7c,e), and diastolic tension increased to 50 ± 23% for CTX-1 and 35 ± 17% for CTX-2 (Figure 7d,f; these figures are similar to Figure 5 and Figure 6 and are shown again here for clarity). After washing, the contraction force was restored to 67 ± 25% for CTX-1 and 66 ± 24% for CTX-2 of the control level and the diastolic tension decreased to 30 ± 18% and 15 ± 3%, respectively. Thus, the PM pretreatment with 2-APB prevented the development of irreversible contracture in the presence of CTX-1 and CTX-2. Our results showed for the first time that SOCE may contribute to the development of CaTx effects in myocardial tissue.

Taken together, our results indicate that influx of extracellular Ca^2+^ plays significant role in the CaTx-mediated pathological effects on PM. Moreover, to the best of our knowledge, it is the first report on the importance of store-operated calcium entry in CaTx-mediated effects. Blocking intracellular Ca^2+^ release from SR by ryanodine had a moderate influence on CaTx effects, preventing transient increase in PM contraction force but had no effect on the development of PM contracture induced either by CTX-1 or CTX-2.

### 2.5. Molecular Mechanisms of the Aorta Rings Contractile Response Induced by Cardiotoxins

Ca^2+^ influx plays an important role in the function of vascular smooth muscle, and intracellular Ca^2+^ concentration determines their contractile state [37]. CaTxs increase intracellular Ca^2+^ concentration, producing a contractile response. Here, we studied the contribution of several Ca^2+^ transport systems to the contraction induced by CaTxs. As shown above (Figure 2), CTX-1 is weaker than CTX-2 when acting on the aorta; therefore, to investigate the effects of the inhibitors, we chose concentrations of 7.5 and 3 µM for CTX-1 and CTX-2, respectively.

**Figure 8:**
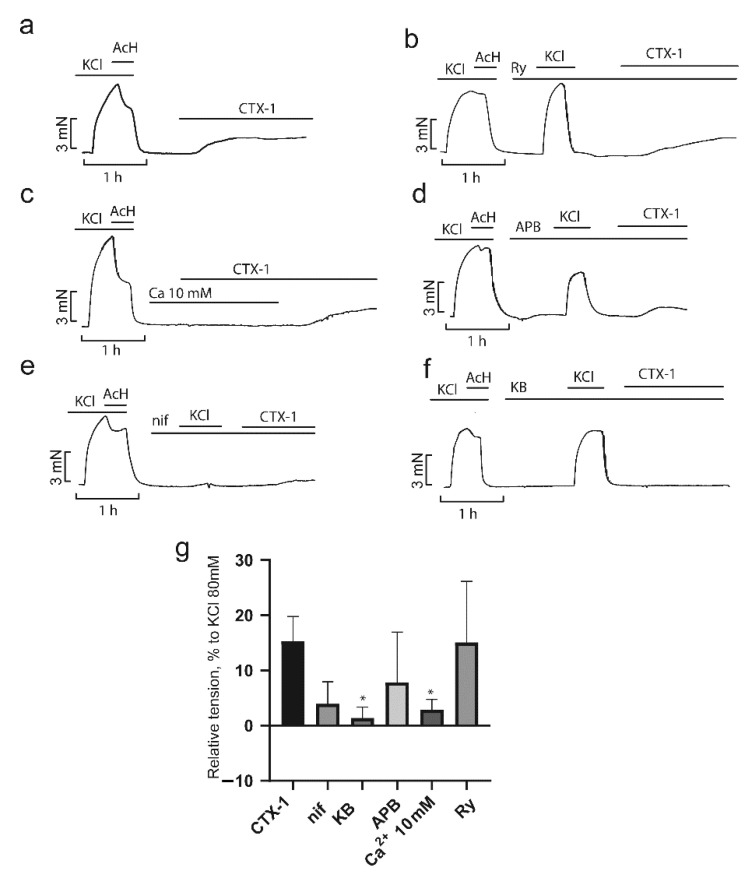
Influence of inhibitors of Ca^2+^ transport mechanisms on the CTX-1 effects in aorta rings (ARs). Typical recordings of the CTX-1 effects on the contractility of the ARs in the absence ((**a**), *n* = 6) and in the presence of inhibitors (**b**–**f**) are shown. (**g**) The force of contraction normalized to that obtained in response to 80 mM KCl. Data are presented as means ± standard error of the mean. * *p* < 0.05 compared to 80 mM KCl. Ry, ryanodine (10 µM, *n* = 3); nif, nifedipine (2 µM, *n* = 3); KB, KB-R7943 (10 µM, *n* = 3); APB, 2-aminoethoxydiphenyl borate (40 µM, *n* = 5); Ca, 10 mM Ca^2+^ (*n* = 3).

**Figure 9:**
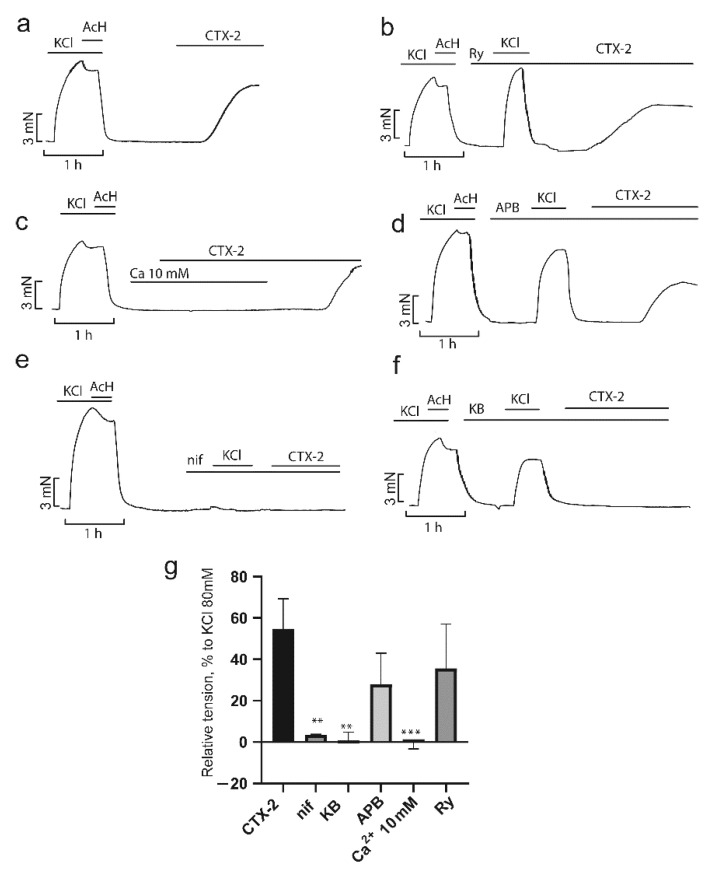
Influence of inhibitors of Ca^2+^ transport mechanisms on the CTX-2 effects in aorta rings (ARs). Typical recordings of the CTX-2 effects on the contractility of the ARs in the absence ((**a**), *n* = 7) and in the presence of inhibitors (**b**–**f**) are shown. (**g**) The force of contraction normalized to that obtained in response to 80 mM KCl. Data are presented as means ± standard error of the mean. ** *p* < 0.01 and *** *p* < 0.005 compared to 80 mM KCl. Ry, ryanodine (10 µM, *n* = 4); nif, nifedipine (2 µM, *n* = 3); KB, KB-R7943 (10 µM, *n* = 3); APB, 2-aminoethoxydiphenyl borate (40 µM, *n* = 4); Ca, 10 mM Ca^2+^ (*n* = 3).

As shown in Figure 8a and Figure 9a, application of CTX-1 and CTX-2 at 7.5 and 3 µM, respectively, resulted in a contractile response corresponding to 12 ± 2 and 50 ± 7%, respectively, of the contraction produced by 80 mM KCl (Figure 8g and Figure 9g). A high concentration of Ca^2+^ ions completely blocked the effect of both toxins (Figure 8c and Figure 9c). The L-type Ca^2+^ channel blocker nifedipine (2 μM) reduced the developed contraction to 3 ± 2 and 3 ± 0% for CTX-1 and CTX-2, respectively, which is in good agreement with earlier data [21,22] (Figure 8e,g and Figure 9e,g). KB-R7943 (10 µM), a reverse-mode NCX inhibitor, significantly reduced the contractility induced by cardiotoxins to 1.4 ± 1.1% and 0.9 ± 2% for CTX-1 and CTX-2, respectively (Figure 8f,g and Figure 9f,g).

Unexpectedly, 2-APB, an inhibitor of SOCE that plays a significant role in the contraction of smooth muscle cells [38], did not have a significant effect on the contraction produce by toxins, which in the presence of 2-APB (40 µM) were equal to 7.8 ± 4.1% and 32 ± 8.8 for CTX-1 and CTX-2, respectively (Figure 8d,g and Figure 9d,g). It has been shown previously that SK&F 96365, another SOCE blocker, prevents contractions caused by cobra cardiotoxin [21]. The difference between the results of this study and ours may be explained by both the different specificity of the inhibitors [39] and the different cardiotoxin concentrations used. Further, we showed that ryanodine, the blocker of Ca^2 +^ release from SR, did not affect the magnitude of the cytotoxin effects at 10 μM; the contractions were equal 15 ± 6.4 and 30.4 ± 8.4% for CTX-1 and CTX-2, respectively (Figure 8b,g and Figure 9b,g). Our data confirm the conclusion [22] about the predominant role of the extracellular Ca^2+^ influx in the development of CaTx-induced contraction. This conclusion is in agreement with earlier work on neonatal rat cardiomyocytes which showed that the use of L-type Ca2^+^ channel blockers prevented contracture development [35]. It seems that, in smooth muscle cells, upon inhibition of Ca^2+^ current, there is no large-scale release of Ca^2+^ from the SR in response to depolarization.

## 3. Conclusions

A comparison of the effects of two types of CaTxs on the rat heart muscle and aorta showed that the P-type toxin (CTX-2) produced stronger effects than the S-type toxin (CTX-1). Both toxins exerted resembling impacts on the contractile properties of the myocardium and tonic contractions of the aortic rings, as well as on the development of effects at the inhibition of calcium current or at the increase of the extracellular calcium concentration. It was demonstrated that the positive segment of the force–frequency relationship in the range of physiological frequencies is the most toxin-sensitive component in the regulation of calcium homeostasis in the rat myocardium. 

Although we found a difference in the effects of S- and P-type toxins, only one toxin of each type was used in this work. To find out whether the differences we observed extend to other toxins, we plan to perform a study on a larger number of CaTxs of different types. Another extension of this work will be comparative study of the effects of S- and P-type CaTxs on endothelium-dependent transient AR relaxation.

By applying various inhibitors of Ca^2+^ channels and exchangers, it was found that several molecular mechanisms leading to increase of intracellular Ca^2+^ concentration may be involved in the effects produced by CaTxs in PM and AR. The most pronounced effect was found at the inhibition of SOCE by 2-APB which strongly attenuated CaTx effects in PM. This is the first indication of SOCE participation in the adverse effects of CaTxs on myocardial contractility. We also found that nifedipine, the L-type Ca^2+^ channel blocker, and KB-R7943, a reverse-mode NCX inhibitor, significantly reduced the AR contraction induced by CaTxs. While the effect of nifedipine has been observed in several works, the effect of the reverse-mode NCX inhibitor on CaTx-induced AR contraction had not previously been demonstrated. Therefore, we showed for the first time the involvement of reverse-mode NCX in the effect of CaTx on the rat aorta. 

## 4. Materials and Methods

### 4.1. Materials

Cardiotoxins were isolated from *Naja oxiana* cobra venom as described earlier [40,41]. Ryanodine and KB-R7943 were from Tocris Bioscience (Bristol, UK). Nifedipine, 2-aminoethoxydiphenyl borate, inorganic salts, and glucose were obtained from Merck KGaA (Darmstadt, Germany). All other reagents obtained from local suppliers were of analytical grade or higher purity.

### 4.2. Animal Handling

Adult male Wistar rats (200–220 g body weight) were used for the experiments. This study did not involve endangered or protected species and was performed in accordance with Directive 2010/63/EU of the European Parliament. All experimental procedures were approved by the Biological Safety and Ethics Committee of the Institute of Cell Biophysics (instruction for the use of laboratory animals in the Institute of Cell Biophysics NO. 57 of 30 December 2011).

### 4.3. Contractility of Papillary Muscles

Isolation of right ventricle papillary muscles (PMs, Figure S1a) was performed from the hearts of anesthetized rats. Measurements of the isometric force of PM contraction were performed in oxygenated (95% O_2_/5% CO_2_) Tyrode solution containing (in mM): NaCl, 135; KCl, 4; MgCl_2_, 1; CaCl_2_, 1.8; NaHCO_3_, 13.2; NaH_2_PO_4_, 1.8; glucose, 11; (pH 7.4) as previously described [42]. In brief, isolated PMs were mounted horizontally in a temperature-controlled chamber (30 ± 0.1 °C), and stretched to a length at which tension of contraction was maximal (Figure S1c). Stimuli were applied using bipolar Ag–AgCl electrodes by square-wave pulses of 5 ms duration and amplitude set at 25% above the excitation threshold. Prior to each experiment, muscle preparations were stimulated at 0.3 Hz for 1 h until complete mechanical stabilization. The following parameters were recorded: the force of contraction, the maximum transient increase in the force of contraction in response to the action of CaTx, and the level of peak diastolic tension (contracture caused by CaTx). The course of the experiment is schematically illustrated in Figure 1a. 

### 4.4. Contractility of Aortic Rings

Aortas were isolated from anesthetized rats, placed in Tyrode solution (similar to that used for PM but with 2.5 mM CaCl_2_) and cleaned of fat and loose connective tissues. Rings (2–3 mm) were cut (Figure S1b). The aortic rings (ARs) were mounted on to two tungsten wires. One wire was fixed to the organ bath wall and the other was connected to a force transducer to record isometric tension (Figure S1d). The temperature of Tyrode solution circulating in the tissue bath was maintained at 30 °C. After establishing the initial load of 2 g, the ARs were adapted for 60 min. At the end of this period the tension on the rings was taken as resting tension. To induce contraction, all rings were exposed to isotonic depolarizing solution (with equimolar replacement of NaCl to obtain 80 mM KCl in solution), and then after 20 min, 10 µM acetylcholine was added to check endothelium integrity. After that, acetylcholine was washed out until the initial tension level was reached (usually 40–60 min) and the pharmacological inhibitor of Ca^2+^ transporting system under study was introduced into the system. After 20 min, isotonic depolarizing solution was given again. After another round of washing from KCl, CaTx was added to solution alone or in the presence of the studied inhibitor. The course of experiment is schematically illustrated on Figure 1b.

### 4.5. Data Analysis and Statistics

Student’s *t*-test was used to compare continuous variables. One-way ANOVA with Dunnett’s post hoc test was used for multiple groups comparison. *p*-value < 0.05 was predetermined as a statistically significant difference. All data are presented as mean ± standard error (S.E.).

## Data Availability

All data obtained in this study are contained within the article.

## Supplementary Materials

The following supporting information can be downloaded at: https://www.mdpi.com/article/10.3390/toxins14020088/s1, Figure S1: Illustration of papillary muscle and aorta ring preparations and schemes of experimental set-ups. (a) Papillary muscle preparation with a fragment of heart septum; (b) aorta ring preparation; (c) scheme of experimental chamber with papillary muscle; (d) scheme of experimental chamber with aorta ring.

## References

[1] Chaisakul J., Isbister G.K., Konstantakopoulos N., Tare M., Parkington H.C., Hodgson W.C. (2012). In vivo and in vitro cardiovascular effects of Papuan taipan (*Oxyuranus scutellatus*) venom: Exploring ”sudden collapse”. Toxicol. Lett..

[2] Vuong N.T., Jackson T.N.W., Wright C.E. (2021). Role of Phospholipases A2 in Vascular Relaxation and Sympatholytic Effects of Five Australian Brown Snake, Pseudonaja spp., Venoms in Rat Isolated Tissues. Front. Pharmacol..

[3] Kini R.M., Koh C.Y. (2020). Snake venom three-finger toxins and their potential in drug development targeting cardiovascular diseases. Biochem. Pharmacol..

[4] Averin A.S., Utkin Y.N. (2021). Cardiovascular Effects of Snake Toxins: Cardiotoxicity and Cardioprotection. Acta Nat..

[5] Kumar T.K., Jayaraman G., Lee C.S., Arunkumar A.I., Sivaraman T., Samuel D., Yu C. (1997). Snake venom cardiotoxins-structure, dynamics, function and folding. J. Biomol. Struct. Dyn..

[6] Lee C.Y., Chang C.C., Chiu T.H., Chiu P.J., Tseng T.C., Lee S.Y. (1968). Pharmacological properties of cardiotoxin isolated from Formosan cobra venom. Naunyn Schmiedebergs Arch. Exp. Pathol. Pharmakol..

[7] Sun J.J., Walker M.J. (1986). Actions of cardiotoxins from the southern Chinese cobra (*Naja naja atra*) on rat cardiac tissue. Toxicon.

[8] Wang H.X., Lau S.Y., Huang S.J., Kwan C.Y., Wong T.M. (1997). Cobra venom cardiotoxin induces perturbations of cytosolic calcium homeostasis and hypercontracture in adult rat ventricular myocytes. J. Mol. Cell. Cardiol..

[9] Gasanov S.E., Shrivastava I.H., Israilov F.S., Kim A.A., Rylova K.A., Zhang B., Dagda R.K. (2015). Naja naja oxiana Cobra Venom Cytotoxins CTI and CTII Disrupt Mitochondrial Membrane Integrity: Implications for Basic Three-Fingered Cytotoxins. PLoS ONE.

[10] Li F., Shrivastava I.H., Hanlon P., Dagda R.K., Gasanoff E.S. (2020). Molecular Mechanism by which Cobra Venom Cardiotoxins Interact with the Outer Mitochondrial Membrane. Toxins.

[11] Dubovskii P.V., Lesovoy D.M., Dubinnyi M.A., Konshina A.G., Utkin Y.N., Efremov R.G., Arseniev A.S. (2005). Interaction of three-finger toxins with phospholipid membranes: Comparison of S- and P-type cytotoxins. Biochem. J..

[12] Wang C.H., Monette R., Lee S.C., Morley P., Wu W.G. (2005). Cobra cardiotoxin-induced cell death in fetal rat cardiomyocytes and cortical neurons: Different pathway but similar cell surface target. Toxicon.

[13] Grishin E.V., Sukhikh A.P., Adamovich T.B., Ovchinnikov Y.A. (1976). Isolation, properties, and amino acid sequence of two cytotoxins from the venom of the Central Asian cobra Naja naja oxiana. Bioorg. Khim..

[14] Cher C.D.N., Armugam A., Zhu Y.Z., Jeyaseelan K. (2005). Molecular basis of cardiotoxicity upon cobra envenomation. Cell. Mol. Life Sci..

[15] Harvey A.L., Kornisiuk E., Bradley K.N., Cerveñansky C., Durán R., Adrover M., Sánchez G., Jerusalinsky D. (2002). Effects of muscarinic toxins MT1 and MT2 from green mamba on different muscarinic cholinoceptors. Neurochem. Res..

[16] Rajagopalan N., Pung Y.F., Zhu Y.Z., Wong P.T.H., Kumar P.P., Kini R.M. (2007). Beta-cardiotoxin: A new three-finger toxin from *Ophiophagus hannah* (king cobra) venom with beta-blocker activity. FASEB J..

[17] Rouget C., Quinton L., Maïga A., Gales C., Masuyer G., Malosse C., Chamot-Rooke J., Thai R., Mourier G., de Pauw E. (2010). Identification of a novel snake peptide toxin displaying high affinity and antagonist behaviour for the α2-adrenoceptors. Br. J. Pharmacol..

[18] Averin A.S., Astashev M.E., Andreeva T.V., Tsetlin V.I., Utkin Y.N. (2019). Cardiotoxins from Cobra *Naja oxiana* Change the Force of Contraction and the Character of Rhythmoinotropic Phenomena in the Rat Myocardium. Dokl. Biochem. Biophys..

[19] Huang S.J., Wu C.K., Sun J.J. (1991). Positive inotropic and toxic action of direct lytic factor on isolated working guinea pig hearts. Zhongguo Yao Li Xue Bao.

[20] Loots J.M., Meij H.S., Meyer B.J. (1973). Effects of Naja nivea venom on nerve, cardiac and skeletal muscle activity of the frog. Br. J. Pharmacol..

[21] Ho K.H., Kwan C.Y., Huang S.J., Bourreau J.P. (1998). Dual effect of cobra cardiotoxin on vascular smooth muscle and endothelium. Zhongguo Yao Li Xue Bao.

[22] Kwan C.Y., Kwan T.K., Huang S.J. (2002). Effect of calcium on the vascular contraction induced by cobra venom cardiotoxin. Clin. Exp. Pharmacol. Physiol..

[23] Lin-Shiau S.Y., Huang H.C., Lee C.Y. (1986). A comparison of the actions of cobra cardiotoxin and scorpion toxin II on the guinea-pig taenia coli. Toxicon.

[24] Harvey A.L., Marshall R.J., Karlsson E. (1982). Effects of purified cardiotoxins from the Thailand cobra (*Naja naja siamensis*) on isolated skeletal and cardiac muscle preparations. Toxicon.

[25] Chen K.M., Guan Y.Y., Sun J.J. (1993). Effects of direct lytic factors from southern Chinese cobra venom on Ca2+ movement in rabbit aorta strip. Zhongguo Yao Li Xue Bao.

[26] Huang S.J., Kwan C.Y. (1996). Inhibition by multivalent cations of contraction induced by Chinese cobra venom cardiotoxin in guinea pig papillary muscle. Life Sci..

[27] Chong H.P., Tan K.Y., Tan C.H. (2020). Cytotoxicity of Snake Venoms and Cytotoxins From Two Southeast Asian Cobras (*Naja sumatrana*, *Naja kaouthia*): Exploration of Anticancer Potential, Selectivity, and Cell Death Mechanism. Front. Mol. Biosci..

[28] Huang J.L., Trumble W.R. (1991). Cardiotoxin from cobra venom affects the Ca-Mg-ATPase of cardiac sarcolemmal membrane vesicles. Toxicon.

[29] Nayler W. (1976). The effect of a cardiotoxic component of the venom of the Indian cobra (*Naja nigricollis*) on the subcellular structure and function of heart muscle. J. Mol. Cell. Cardiol..

[30] Monteiro D.A., Kalinin A.L., Selistre-de-Araujo H.S., Vasconcelos E.S., Rantin F.T. (2016). Alternagin-C (ALT-C), a disintegrin-like protein from Rhinocerophis alternatus snake venom promotes positive inotropism and chronotropism in fish heart. Toxicon.

[31] Endoh M. (2004). Force-frequency relationship in intact mammalian ventricular myocardium: Physiological and pathophysiological relevance. Eur. J. Pharmacol..

[32] Stuyvers B.D., McCulloch A.D., Guo J., Duff H.J., ter Keurs H.E.D.J. (2002). Effect of stimulation rate, sarcomere length and Ca(2+) on force generation by mouse cardiac muscle. J. Physiol..

[33] Bers D.M. (2000). Calcium fluxes involved in control of cardiac myocyte contraction. Circ. Res..

[34] Fletcher J.E., Tripolitis L., Beech J. (1993). Species difference in modulation of calcium release by *Naja naja kaouthia* snake venom cardiotoxin in terminal cisternae from human and equine skeletal muscle. Toxicon.

[35] Tzeng W.F., Chen Y.H. (1988). Suppression of snake-venom cardiotoxin-induced cardiomyocyte degeneration by blockage of Ca2+ influx or inhibition of non-lysosomal proteinases. Biochem. J..

[36] Debnath A., Saha A., Gomes A., Biswas S., Chakrabarti P., Giri B., Biswas A.K., Gupta S.D., Gomes A. (2010). A lethal cardiotoxic-cytotoxic protein from the Indian monocellate cobra (*Naja kaouthia*) venom. Toxicon.

[37] Brozovich F.V., Nicholson C.J., Degen C.V., Gao Y.Z., Aggarwal M., Morgan K.G. (2016). Mechanisms of Vascular Smooth Muscle Contraction and the Basis for Pharmacologic Treatment of Smooth Muscle Disorders. Pharmacol. Rev..

[38] Avila-Medina J., Mayoral-Gonzalez I., Dominguez-Rodriguez A., Gallardo-Castillo I., Ribas J., Ordoñez A., Rosado J.A., Smani T. (2018). The Complex Role of Store Operated Calcium Entry Pathways and Related Proteins in the Function of Cardiac, Skeletal and Vascular Smooth Muscle Cells. Front. Physiol..

[39] Ishida H., Saito S.-Y., Hishinuma E., Ishikawa T. (2017). Differential Contribution of Nerve-Derived Noradrenaline to High K+-Induced Contraction Depending on Type of Artery. Biol. Pharm. Bull..

[40] Dubovskii P.V., Lesovoy D.M., Dubinnyi M.A., Utkin Y.N., Arseniev A.S. (2003). Interaction of the P-type cardiotoxin with phospholipid membranes. Eur. J. Biochem..

[41] Dubovskii P.V., Dubinnyi M.A., Volynsky P.E., Pustovalova Y.E., Konshina A.G., Utkin Y.N., Arseniev A.S., Efremov R.G. (2018). Impact of membrane partitioning on the spatial structure of an S-type cobra cytotoxin. J. Biomol. Struct. Dyn..

[42] Nakipova O.V., Averin A.S., Evdokimovskii E.V., Pimenov O.Y., Kosarski L., Ignat’ev D., Anufriev A., Kokoz Y.M., Reyes S., Terzic A. (2017). Store-operated Ca2+ entry supports contractile function in hearts of hibernators. PLoS ONE.

