# Supplementary material for: Effects of Cardiotoxins from Naja oxiana Cobra Venom on Rat Heart Muscle and Aorta: A Comparative Study of Toxin-Induced Contraction Mechanisms
Source: Toxins (Basel). 2022 Jan 24;14(2):88. doi: 10.3390/toxins14020088 (PMC8878657; doi:10.3390/toxins14020088)
Supplement: Supplementary file 1 [file toxins-14-00088-s001.zip › toxins-1543262-supplementary.pdf]

Supplementary Materials: Effects of Cardiotoxins from *Naja oxiana* Cobra Venom on Rat Heart Muscle and Aorta: A Comparative Study of Toxin-Induced Contraction Mechanisms

Alexey S. Averin, Miroslav N. Nenov, Vladislav G. Starkov, Victor I. Tsetlin and Yuri N. Utkin

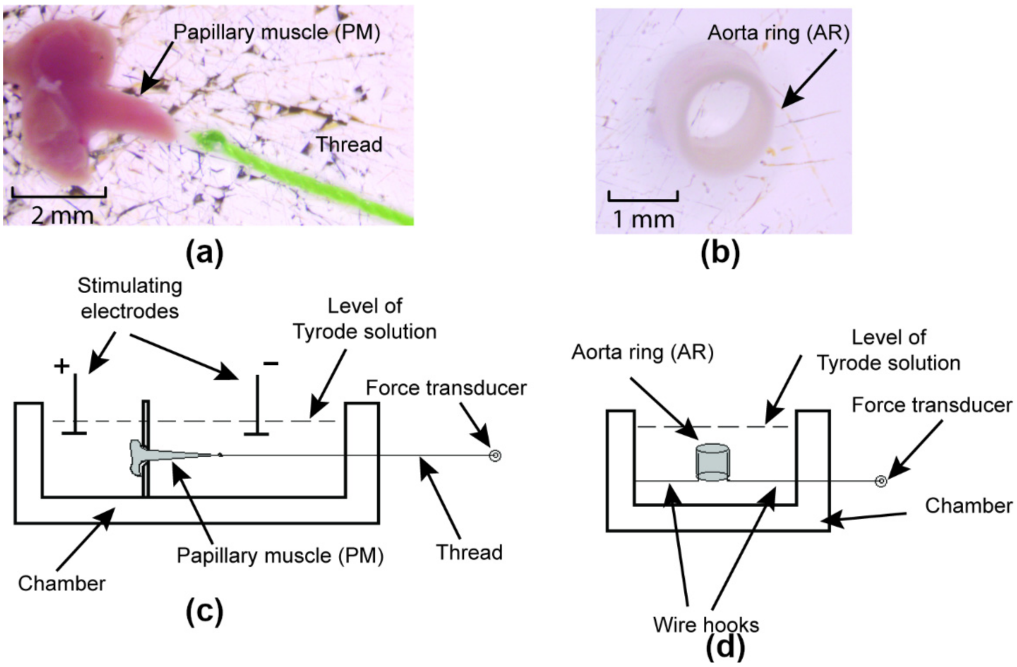

Figure S1. Illustration of papillary muscle and aorta ring preparations and schemes of experimental set-ups. (a) Papillary muscle preparation with a fragment of heart septum; (b) aorta ring preparation; (c) scheme of experimental chamber with papillary muscle; (d) scheme of experimental chamber with aorta ring.
